# Editorial: Biogenic amines in foods

**DOI:** 10.3389/fmicb.2015.00472

**Published:** 2015-05-18

**Authors:** Giovanna Suzzi, Sandra Torriani

**Affiliations:** ^1^Faculty of BioScience and Technology for Food, Agriculture and Environment, University of TeramoTeramo, Italy; ^2^Department of Biotechnology, University of VeronaVerona, Italy

**Keywords:** biogenic amines, histamine, tyramine, sausages, fish, cheeses, wine

Biogenic amines (BA) are nitrogenous compounds of low molecular weight and are essential at low concentrations for natural metabolic and physiological functions in animals, plants, and microorganisms. Histamine, putrescine, cadaverine, tyramine, tryptamine, 2-phenylethylamine, spermine and spermidine are the most important BA in foods in which they are mainly produced by microbial decarboxylation of amino acids. Many factors influence BA production in foods, including food physico-chemical parameters (NaCl, pH and ripening temperature), storage, and distribution conditions, manufacturing processes and practices, presence of decarboxylase-positive microorganisms, raw material quality, and availability of free amino acids (Linares et al., 2012). Nonetheless, consumption of food or beverages containing high amounts of these compounds can have toxic effects such as hypertension, cardiac palpitations, headache, nausea, diarrhea, flushing, and localized inflammation; in extreme cases the intoxication may have fatal outcome. The degree of BA intoxication depends on the amount and type of BA ingested and the correct functioning of the detoxification system. In fact, after food consumption, small quantities of BA are commonly metabolized in the human gut to physiologically less active forms through the activity of the amine oxidizing enzymes, monoamine and diamine oxidases. So the toxic level of BA ingested is difficult to establish, as this depends on the individual sensitivity and health status of consumers. Moreover the malfunction or reduced activity of amine oxidase can result in high BA blood levels, whereas people taking drugs with amino oxidase inhibitor and/or alcohol show interaction with the detoxification system.

Among intoxications related with BA there is the “Scombroid poisoning” caused by histamine which is the only BA with regulatory limits, set by European Commission, up to a maximum of 200 mg/kg in fresh fish and 400 mg/kg in fishery products treated by enzyme maturation in brine (Visciano et al., 2012, 2014). After fish, cheese is the next most commonly implicated food item associated with tyramine poisoning, so called “Cheese reaction,” related with its high content in aged cheeses (Schirone et al., 2012). Other potentially BA, specially histamine and putrescine are also present in milk-based fermented foods (Linares et al., 2012).

Moreover in fermented beverages, such as wine, it is very difficult to minimize content of BA, that are produced mainly through the decarboxylation of amino acids by yeasts during fermentation and/or lactic acid bacteria during malolactic fermentation. In particular vintage, grape variety, geographical region, and vinification methods such as grape skin maceration are some of the variables that can lead to an increase of precursor amino acids and subsequently the BA content in wine (Smit et al., 2012). Recently, some *Lactobacillus plantarum* strains isolated from wine and other oenological source were tested for their ability to degrade BA. Two strains were selected for their potential ability to reduce BA in wine (putrescine and tyramine) and to design malolactic starter cultures (Capozzi et al., 2012).

Among the approaches useful to control the formation of BA, such as the reduction of microbial growth through chilling and freezing or hydrostatic pressures, irradiation, controlled atmosphere packaging, or the use of food additives, etc., the use of selected starter cultures free of the potential to form BA, has been proposed as one of the best technological measures to control aminogenesis during traditional sausages production (Latorre-Moratalla et al., 2012). In fact in traditional dry sausages high content of BA can be produced by different microbial groups such as lactic acid bacteria and enterococci, but also by staphylococci and bacilli (Bermúdez et al., 2012).

Among the food BA, polyamines are ubiquitous substances considered to be bioregulators of numerous cells functions and are involved in tissue repair and in intracellular signaling. Although many biological functions have been attributed to polyamines, high levels of these compounds in foodstuffs can have toxicological effects; however, no safe level for the intake of polyamines in a diet as yet been established. The polyamine agmatine, derived from arginine, is present at high levels in alcoholic beverages, such as wine, beer, sake (Galgano et al., 2012).

The articles within this eBook address various issues related to the qualitative and quantitative presence of BA in cheese, dry sausages, wine, and fish. The possible inactivation and scavenging of these compounds by technological processes and amine oxidase activity of some microorganisms is also reported.

## Conflict of interest statement

The authors declare that the research was conducted in the absence of any commercial or financial relationships that could be construed as a potential conflict of interest.

## References

[B1] Bermúdez R. Lorenzo J. M. Fonseca S. Franco I. Carballo J. (2012). Strains of *Staphylococcus* and *Bacillus* isolated from traditional sausages as producers of biogenic amines. Front. Microbiol. 3:151. 10.3389/fmicb.2012.00151 22529847 PMC3328851

[B2] Capozzi V. Russo P. Ladero V. Fernández M. Fiocco D. Alvarez M. A.. (2012). Biogenic amines degradation by *Lactobacillus plantarum*: toward a potential application in wine. Front. Microbiol. 3:122. 10.3389/fmicb.2012.00122 22485114 PMC3316997

[B3] Galgano F. Caruso M. Condelli N. Favati F. (2012). Focused review: agmatine in fermented foods. Front. Microbiol. 3:199. 10.3389/fmicb.2012.00199 22701114 PMC3369198

[B4] Latorre-Moratalla M. L. Bover-Cid S. Veciana-Nogués M. T. Vidal-Carou M. C. (2012). Control of biogenic amines in fermented sausages: role of starter cultures. Front. Microbiol. 3:169. 10.3389/fmicb.2012.00169 22586423 PMC3345612

[B5] Linares D. M. del Río B. Ladero V. Martínez N. Fernández M. Martín M. C.. (2012). Factors influencing biogenic amines accumulation in dairy products. Front. Microbiol. 3:180. 10.3389/fmicb.2012.00180 22783233 PMC3390585

[B6] Schirone M. Tofalo R. Visciano P. Corsetti A. Suzzi G. (2012). Biogenic amines in Italian Pecorino cheese. Front. Microbiol. 3:171. 10.3389/fmicb.2012.00171 22586425 PMC3347038

[B7] Smit A. Y. Engelbrecht L. du Toit M. (2012). Managing your wine fermentation to reduce the risk of biogenic amine formation. Front. Microbiol. 3:76. 10.3389/fmicb.2012.00076 22419915 PMC3301445

[B8] Visciano P. Schirone M. Tofalo R. Suzzi G. (2012). Biogenic amines in raw and processed seafood. Front. Microbiol. 3:188. 10.3389/fmicb.2012.00188 22675321 PMC3366335

[B9] Visciano P. Schirone M. Tofalo R. Suzzi G. (2014). Histamine poisoning and control measures in fish and fishery products. Front. Microbiol. 5:500. 10.3389/fmicb.2014.00500 25295035 PMC4172148

